# Interaction between visual working memory and upright postural control in young adults: an event-related potential study based on the n-back paradigm

**DOI:** 10.3389/fnins.2024.1387865

**Published:** 2024-06-26

**Authors:** Sharui Shan, Feng Hong, Liyan Cui, Chenming Sun, Jianliang Lu, Zhuoming Chen, Wenwen Cheng

**Affiliations:** ^1^Department of Rehabilitation, The First Affiliated Hospital of Guangdong Pharmaceutical University, Guangzhou, China; ^2^Department of Rehabilitation, The First Affiliated Hospital of Jinan University, Guangzhou, China; ^3^Department of Rehabilitation, Guangzhou University of Chinese Medicine, Guangzhou, China; ^4^Department of Neurology, Maoming People’s Hospital, Maoming, China

**Keywords:** working memory, n-back paradigm, event-related potential, dual-task paradigm, upright posture

## Abstract

As a part of the overall information-processing system of the brain, postural control is related to the cognitive processes of working memory. Previous studies have suggested that cognitive tasks and postural control processes can compete for resources in common brain areas, although there is an “inverted U” relationship between arousal level and behavioral control – the arousal level of individuals changes when performing cognitive tasks. However, the exact neural connections between the two are unclear. This may be related to the nature of cognitive tasks. Some studies believe that posture occupies not only spatial information processing resources but also visual non-spatial information processing resources. Other studies believe that posture control only occupies spatial information processing resources in the central system, but does not occupy non-spatial information processing resources. Previous studies used different cognitive task materials and reached different conclusions. In this study, we used the same visuospatial and non-spatial materials, the n-back visual working memory paradigm, the event-related potential technique to investigate the effects of visuospatial and non-spatial working memory tasks on adolescents’ postural control under different cognitive loads. The results of this study showed that in both visuospatial and non-spatial conditions, the N1 effect of the parieto-occipital lobe was larger during upright posture than in the sitting position (160–180 ms), the P300 effect of the central parieto-occipital region (280–460 ms) was induced by working memory in different postures, and the P300 wave amplitude was higher in the sitting position than in the upright position. We demonstrated that upright postural control enhances early selective attention but interferes with central memory encoding, thus confirming that postural control and visuospatial and non-spatial working memory share brain regions and compete with each other.

## 1 Introduction

Postural control refers to when the position of the body in space is controlled to achieve the purposes of stability and orientation. Postural control is achieved through interactions between individuals, their actions, and the surrounding environment (Woollacott et al., 2001). In general, postural control refers to the maintenance of an upright posture and a stable center of gravity (Watson, 1999). Previous studies have shown that the control of upright posture balance is related to the vestibular, proprioceptive, and visual systems (Allum and Keshner, 1986; Ozdemir et al., 2017) and is associated with cognitive function, especially working memory. As vision provides the most intuitive spatial cues for posture control, and experiments show that postural sway increases by 8 more than 50% when vision is lost, this sense is considered to play a predominant role in upright balance posture control (Collins and De Luca, 1995; Ren et al., 2010).

At present, relevant theories of spatial memory and non-spatial memory cognitive task and postural control abilities can be roughly divided into two types: the competition model theory and U-shaped nonlinear interaction model theory. According to the competition model theory, interference between upright posture control and certain cognitive tasks occurs because they compete for common central processing resources; if there is no interference, it is because their processing centers utilize different processing resources (Fraizer and Mitra, 2008). Kerr et al. (1985) first adopted the dual-task paradigm, in which all subjects performed two different cognitive tasks while maintaining a stable upright posture. The first involves spatial memory (memory of the spatial position of text within a picture) and the second non-spatial memory (word memory). They concluded that the control of upright balance posture (1) is not an automatic process and (2) occupies spatial information processing resources in the central system, but not non-spatial information processing resources. However, further studies found that non-spatial cognitive tasks, such as reaction time tasks and mental counting tasks, can also interfere with the stability of upright balance postures (Huxhold et al., 2006; Schmid et al., 2007; Maylor et al., 2011). Using behavioral data and functional near-infrared spectroscopic imaging, researchers have demonstrated selective interactions between upright balance postural control and working memory and more pronounced interactions between upright balance postural control and spatial working memory (Chen et al., 2018). However, previous studies found that participants’ upright posture stability was improved when performing cognitive tasks (Mylene et al., 2001; Andersson et al., 2002; Kataoka et al., 2007), and the authors proposed that cognitive tasks may be mediated by the participant’s arousal levels, affecting posture control. The arousal level of individuals changes when performing cognitive tasks, and there is an “inverted U” relationship between arousal level and behavioral control.

A number of researchers have adopted event-related potential (ERP) technology and found that N1 (negative potential around 100 ms after stimulus onset) components related to the sensory input processing of postural control were induced by Cz electrodes in the central region during disturbed postural control (Quant et al., 2004; Harada et al., 2009; Little and Woollacott, 2015; Varghese et al., 2019). However, in the dual-task state (in which the cognitive task was to visually track an object), the amplitude of the N1 component decreased significantly. The participants’ level of attention was positively correlated with the amplitude of N1, which could be used as an objective indicator of perceptual formation of attention (Finnigan et al., 2011). The amplitude of P300 (positive displacement occurring around 300 ms after stimulus) reflects the allocation of attention resources, and its latency reflects the speed or efficiency of the brain’s processing of cognitive tasks. Initially, it was found that, when studying the ERP of typical working memory, the amplitude of P300 increased with increased task load, and its latency was also influenced by task load and perceptual complexity (Mccarthy and Donchin, 1981; Kok, 1997; Brown et al., 2007).

Previous studies have used highly diverse stimulus materials, for example, graphics and speech stimuli respectively representing space and non-space, and have not taken into account the cognitive load. Therefore, the different cognitive upright paradigms produced different results in the studies. In the n-back task, the subjects are tasked with making “consistent” judgments of target features, such as English letters, spatial orientation, facial identity, expression, etc. The parameter N is a key variable in the n-back paradigm, and many studies have investigated the impact of cognitive load on working memory updates by changing N (Redick and Lindsey, 2013). However, there are few reports of researchers studying visual working memory and upright posture control using the n-back paradigm.

This study adopted the n-back visual working memory paradigm to investigate the effects of working memory tasks under different cognitive loads on upright balance postural control. The direct relationship between working memory and postural control was studied with the ERP technique. In addition to studying whether spatial and non-spatial working memory and upright balance postural control utilize common brain regions, we also observed whether different cognitive loads interact with each other under different postural positions.

## 2 Methods and materials

### 2.1 General information

The sample size of this study was determined based on previous ERP trial studies and the results collected by Clayson et al. (2019). Fifteen participants were required for paired *T*-test and 11 participants in each group were required for repeated analysis of variance (ANOVA) (Clayson et al., 2019). A total of 25 healthy subjects from Jinan University were recruited for the experimental group of this study, from all of whom, we obtained informed consent. After ethical review, five subjects were excluded due to failure to complete the whole experiment. In total, 20 volunteers participated, including 11 males and 9 females, ranging in age from 18 to 26 years. The mean age (± SE) was 21.8 (±0.54) years; height range was 150–175 cm; average height (±SE) was 165.05 (±1.67); and their body mass index (BMI) ranged from 18.9 to 23.10 kg/m^2^, with an average BMI (± SE) of 21.05 (±0.27) kg/m^2^. After the study, all subjects received corresponding compensation. All volunteers were right-handed, native Chinese speakers, had no history of neurological diseases or head injury, had normal vision or corrected vision, and no other special abnormalities. Subjects who could not stand with their feet together for 2.5–3 min were excluded.

### 2.2 Stimulus materials and subjects’ tasks

**FIGURE 1:**
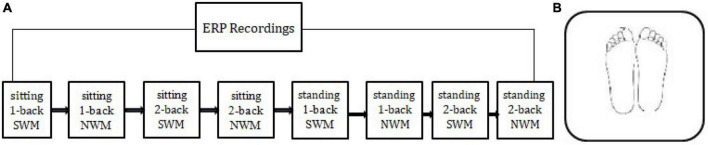
**(A)** Experimental flow chart. The experiment was divided into eight block. SWM was visuo-spatial working memory, NWM was non-spatial working memory. All participants could randomly choose the order in which to conduct the experiment. **(B)** Upright posture controlled standing. Subjects stood upright with feet completely together.

Stimuli were presented on a computer screen (resolution of 1,024 × 768 pixels and refresh rate of 85 Hz) positioned 1 m from the eyes of the subject. Both the horizontal and vertical viewing angles were approximately 8.5°. While avoiding some letters with similar forms, we selected as experimental stimulus material the following six English letters in capital format: A, B, E, Q, G, and J. The experimental stimulus was presented in one of four possible positions on the screen, up, down, left, or right, equidistant apart and orientated around a central “+.”The target stimulus was presented at 500 ms, and the interval was 2,500 ms. The experiment was divided into eight blocks involving 1-back and 2-back working memory tasks of different visual functions in two body positions (Figure 1A). Body position was divided into a sitting position and standing position (Figure 1B). During data collection, the subjects randomly chose to sit or stand first through the question bank data. Each block was about 144 s. With 48 experimental trials, including intermediate rest times, the total length of the experiment was 21.5 min.

**FIGURE 2:**
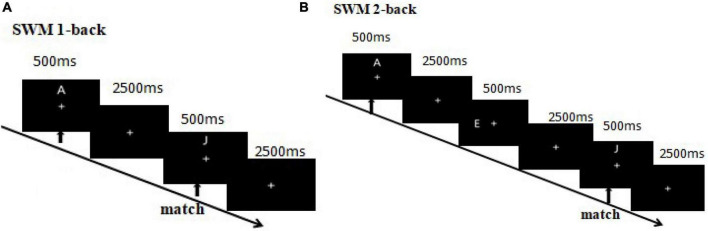
**(A)** 1-Back visuo-spatial working memory. The first arrow indicates the appearance of the first (target) stimulus and the beginning of the task. The second arrow indicates the appearance of the second (probe) stimulus, where participants were required to match the second and first stimuli. Participants needed to match the location of a letter between the second and first trials. A match trial occurred if the second and first letters had matching locations, otherwise it was a mismatch trial. **(B)** 2-Back visuo-spatial working memory. Participants needed to match the location of a letter between the third and first trials. A match trial occurred if the third and first letters had matching locations, otherwise it was a mismatch trial.

**FIGURE 3:**
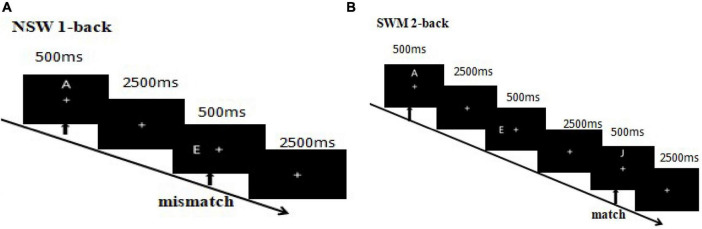
**(A)** 1-Back non-spatial working memory. The first arrow indicates the appearance of the first (target) stimulus and the beginning of the task. The second arrow indicates the appearance of the second (probe) stimulus, where participants were required to match the second and first stimuli. A match trial occurred if the second and first letters matched, otherwise it was a mismatch trial. **(B)** 2-Back non-spatial working memory. Participants were required to match the third and first stimuli. A match trial occurred if the third and first letters matched, otherwise it was a mismatch trial.

Subjects were instructed to press a “match” or “mismatch” button to register their judgment as quickly and accurately as possible. The left and right mouse buttons were to be pressed with the right index and middle fingers. The participants pressed the left mouse button for a match and the right mouse button for a mismatch, with button-response pairing counter-balanced across subjects. The six letters appeared the same number of times in each block, and the probability of matching and mismatching in each block was 1:1. In the visuospatial component, participants were asked to match the location of the target letter and probe letter in each 1-back and 2-back sequence (Figures 2A, B). In the non-spatial working memory component, participants were asked to match the identity of the target letter and probe letter in each 1-back and 2-back sequence (Figures 3A, B).

### 2.3 Electroencephalogram recordings

An electroencephalogram (EEG) was continuously recorded at a sampling rate of 1,000 Hz with a 19-channel EEG amplifier (the Symtop Instrument). The recording bandwidth was 0.5–100 Hz. The international 10–20 system (FP1, FP2, F3, F4, C3, C4, P3, P4, O1, O2, F7, F8, T3, T4, T5, T6, Fz, Cz, and Pz) was used, with linked earlobes as the reference. The electrode impedances were kept below 10 kΩ.

### 2.4 Statistical analysis

#### 2.4.1 Behavioral index analysis data

All data are consistent with normality. The effects of the experimental condition match accuracy, RT (trimmed mean), were determined by multivariate ANOVAs in SPSS 20.0 software. If any main effect or interaction existed, paired sample *T*-tests were used to make further comparisons. The threshold of probability for all analyses was set at 0.05.

#### 2.4.2 ERP data analysis statistical software

Mindwave-sorting and statistical parametric mapping (SPM) were used to analyze ERP space-time (Zhou et al., 2004, 2019; Cao et al., 2017; Cheng et al., 2021), and EEG data were preprocessed using Mindwave-sorting offline classification. First, any artifacts in the eye, muscle, and EEG signals were detected by Mindwave-sorting at a threshold of ±70 μV and automatically corrected by principal component analysis (Lins et al., 1993a,b). Subsequently, a period of 100 ms before the target stimulus to 600 ms after the target stimulus was segmented, and baseline correction was performed to correct for the pre-stimulus activity. The ERP baseline measurement was the average amplitude of the 100-ms pre-stimulus interval. Finally, we obtained the total mean value of the generalized mean waveform execution SPM for eight test types. ERP’s statistical software package was used to run paired *T*-tests corresponding to the ERP of each channel. The results were obtained from the difference values of *F* to obtain the statistical parameter image SPM. The threshold of significance for all analyses was set at 0.05.

## 3 Results

Table 1 shows descriptive statistical results for response time and accuracy. Tables 2, 3 show that there were significant differences in response time among the different task types (*P* = 0.009). There were significant differences among the different task difficulties (*P* < 0.001), but there was no significant interaction among the three task types. The accuracies (*P* = 0.010) and the difficulties (*P* < 0.001) of the different task types were significantly different. Task difficulty and task type had no significant interaction effects on accuracy (*P* = 0.040, *P* > 0.016, Bonferroni correction threshold). As can be seen from Table 4, when a 1-back visuospatial task was performed by volunteers in the upright position, reaction time and accuracy were statistically different from those recorded in the seated position (*P* = 0.001, *P* = 0.017), and the reaction speed and accuracy of the 1-back visuospatial task in the upright position were greater than those in the seated position. In the 1-back visual non-spatial task, the upright position reaction speed was faster (*P* = 0.018), and the difference was statistically significant, but there was no difference in accuracy (*P* = 0.416).

**TABLE 1:** Statistical description results of response time and accuracy (*M* ± SD) (*n* = 20).

	Task type	Task difficulty	RT (MS)	ACC
Upright	Non-spatial	1-Back	671.56 ± 120.85	0.928 ± 0.13
		2-Back	893.32 ± 280.76	0.863 ± 0.12
		Total	782.44 ± 241.10	0.896 ± 0.13
	Spatial	1-Back	725.87 ± 170.22	0.947 ± 0.11
		2-Back	1,008.14 ± 305.07	0.697 ± 0.18
		Total	867.01 ± 282.64	0.822 ± 0.19
Seated	Non-spatial	1-Back	731.43 ± 155.03	0.888 ± 0.17
		2-Back	902.47 ± 282.06	0.817 ± 0.18
		Total	816.95 ± 240.77	0.852 ± 0.18
	Spatial	1-Back	898.90 ± 284.36	0.842 ± 0.15
		2-Back	971.58 ± 267.33	0.756 ± 0.15
		Total	935.24 ± 274.89	0.780 ± 0.16

RT, response time; MS, millisecond; ACC, accuracy.

**TABLE 2:** Results of analysis of variance of three factors for reaction time.

RT	df	*F*	*P*	η^2^
Position	1	1.792	0.183	0.09
Task	1	6.984	0.009*	0.27
Difficulty	1	23.722	<0.001**	0.56
Position × task	1	0.193	0.661	0.01
Position × difficulty	1	2.875	0.092	0.13
Task × difficulty	1	0.061	0.806	0.00
Position × task × difficulty	1	1.071	0.302	0.05

RT, response time.

*P < 0.05,

**P < 0.001.

**TABLE 3:** Results of analysis of variance of three factors for accuracy.

ACC	df	*F*	*P*	η^2^
Position	1	1.867	0.174	0.09
Task	1	6.835	0.010*	0.26
Difficulty	1	23.754	<0.001**	0.56
Position × task	1	0.195	0.660	0.01
Position × difficulty	1	2.639	0.106	0.12
Task × difficulty	1	4.312	0.040	0.18
Position × task × difficulty	1	3.076	0.081	0.14

RT, response time.

*P < 0.05,

**P < 0.001.

**TABLE 4:** Accuracy and reaction time paired *T*-test results.

Pair	*t1* (ACC)	*P1* (ACC)	*t2* (RT)	*P2* (RT)
10–30	2.607	0.017*	-3.875	0.001*
15–35	0.832	0.416	-2.594	0.018*
20–40	-1.539	0.140	0.601	0.555
25–45	1.401	0.177	-0.331	0.745
10–20	6.208	<0.001**	-4.709	<0.001**
15–25	3.077	0.006*	-4.680	<0.001**
30–40	1.887	0.075	-1.625	0.121
35–45	1.258	0.224	-4.159	0.001*
10–15	0.651	0.523	2.363	0.029*
20–25	-3.690	0.002*	2.231	0.038*
30–35	-0.867	0.397	3.661	0.002*
40–45	-1.647	0.116	1.896	0.073

Ten upright spatial 1-back; 15 upright non-spatial 1-back; 20 upright spatial 2-back; 25 upright non-spatial 2-back; 30 seat spatial 1-back; 35 seat non-spatial 1-back; 40 seat spatial 2-back; 45 seat non-spatial 2-back.

*P < 0.05,

**P < 0.001.

### 3.1 Spatiotemporal patterns of average waveform and SPM (*t*) of upright 1-back spatial working memory vs. seated 1-back spatial working memory

#### 3.1.1 Average waveform and component analysis

**FIGURE 4:**
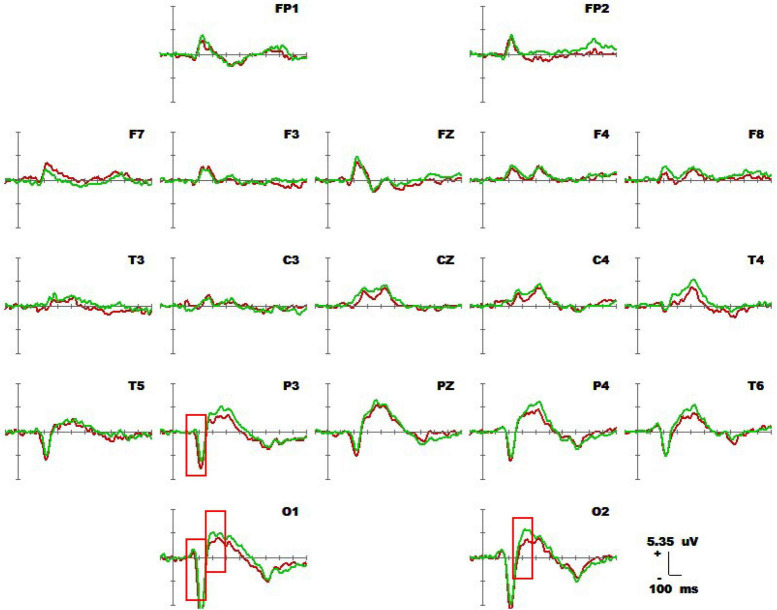
Average waveforms (–100 to 600 ms) across 19 electrodes for 20 subjects performing the visual-spatial 1-back test in upright position (red line) vs. visual-spatial 1-back test in seated position (green line). The baseline of the ERPs is the average amplitude of the waveforms over 100 ms before the stimulus is presented.

Figure 4 shows 1-back spatial working memory in the upright position (red waveform) and 1-back spatial working memory in the seated position (green waveform). There were two stages of differences in waveform between the two groups. The first stage was N1 in the parieto-occipital lobe (160-180 ms), and the amplitude of N1 in the upper position of electrodes placement O1 and P3 was higher than that in the sitting position during this period. The second stage was the occipital lobe (280-360 ms). In this stage, the amplitudes of P300 at electrodes O1 and O2 in the upright position were lower than those in the seated position. The specific statistical data for the amplitude differences between the two ERPs on typical electrodes are shown in Table 5. At the P3 electrode, the maximum effect of N1 was 1.56 μV at 160 ms, −1.73 μV when upright, and −0.17 μV when seated. The maximum expression value of the P300 effect was 2.56 μV at 300 ms at electrode O2, and the corresponding wave amplitudes were 3.82 μV for the upright position and 6.38 μV for the seated position.

**TABLE 5:** Difference in ERP between upright 1-back visuospatial working memory and seated 1-back visuospatial working memory significant waveform effect (*n* = 20).

Effect	N1 (O1)		N1 (P3)		P300 (O2)		P300 (P3)		P300 (O1)	
	Stat	P/wo	Stat	P/wo	Stat	P/wo	Stat	P/wo	Stat	P/wo
*t*/*P*	-2.12	0.047	-2.20	0.040	-2.91	0.009	-2.30	0.032	-2.25	0.036
Cohen’s *d*/WO	-0.973	160	-1.009	160	-1.335	300	-1.055	340	-1.032	280

WO is the time window. The time window was set to 20 ms.

#### 3.1.2 Spatiotemporal patterns of SPM (*t*)

**FIGURE 5:**
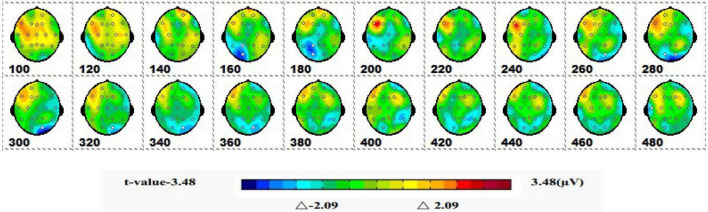
The ERPs of 1-back space working memory in upright position vs. 1-back space working memory in seated position were tested by two-tailed paired *T*-test, and the spatio-temporal pattern of SPM (*t*) (0–600 ms) was obtained by interpolation in average *T*-value. The time window is set at 20 ms. The colors beyond the 0.05 significant threshold (19) = 2.09 at the two ends of the color scale represent significant regions. The white dots represent the electrode sites with significant differences.

Figure 5 shows a topographic map of the SPM (*t*) (0-600 ms) two-tailed paired *T*-test results. The white bright blue locations were divided into thresholds corresponding to *P* = 0.05: *t*(1,19) = ±2.09. The white dots on the topographic map represent electrode sites with significant differences. The 1-back spatial working memory in the upright position vs. 1-back spatial working memory in the sitting position was initially shown in the parieto-occipital lobe at 160-180 ms, and the mean amplitude of 1-back spatial working memory in the upright position was higher than that in the seated position. Then, significant differences again appeared in the occipital lobe at 280–360 ms, and the mean amplitude was higher while sitting than standing.

### 3.2 Spatiotemporal patterns of average waveform and SPM (*t*) of upright 1-back non-spatial working memory vs. seated 1-back non-spatial working memory

#### 3.2.1 Average waveform and component analysis

**FIGURE 6:**
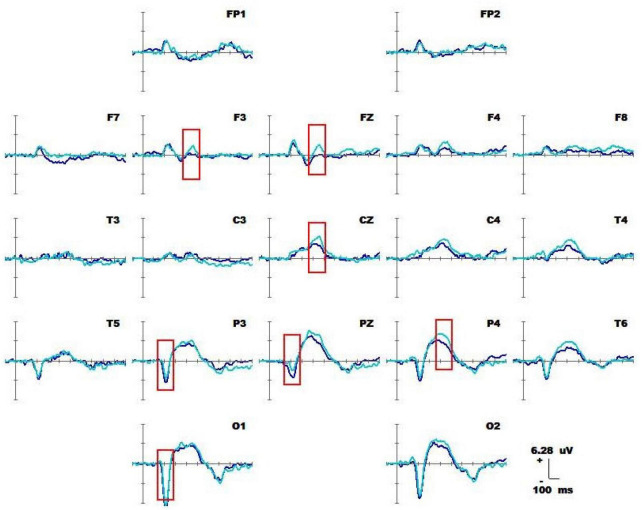
Average waveform of 1-back non-spatial working memory (blue waveform) for 20 subjects in upright position vs. 1-back non-spatial working memory (light blue waveform) at 19 electrodes (–100 to 600 ms). The baseline of the ERPs was the average amplitude of the waveform within 100 ms before the stimulus was presented.

Figure 6 shows the results for 1-back non-spatial working memory in the upright position (blue waveform) and 1-back non-spatial working memory in the seated position (light blue waveform). There were two stages of differences in waveforms between the two groups. The first stage was N1 (160-180 ms) in the parieto-occipital lobe. During this time, the amplitudes of N1 at electrodes O1, P3, and Pz in the standing position were higher than those in the sitting position. The second stage was in the parietal region and central prefrontal region (400-460 ms). At this stage, the amplitudes of P300 at electrodes Fz, F3, P4, Pz, and Cz recorded while the participants were in the upright position were lower than those recorded in the sitting position. The specific statistical data for the amplitude differences between the two ERPs at typical electrodes are shown in Table 6. The maximum N1 effect was 2.42 μV at 180 ms at the Pz electrode, and the corresponding wave amplitude was −4.73 μV in the upright position and −2.31 μV in the seated position. The maximum expression value of the P300 effect was 3.06 μV at 440 ms at the Cz electrode, and the corresponding amplitude was 3.83 μV in the upright position and 6.89 μV in the seated position.

**TABLE 6:** Difference in ERP between upright 1-back visual non-spatial working memory and seated 1-back visual non-spatial working memory showing significant waveform effect (*n* = 20).

Effect	N1 (Pz)		P300 (Fz)		P300 (F3)		P300 (Cz)		P300 (P4)	
	Stat	P/wo	Stat	P/wo	Stat	P/wo	Stat	P/wo	Stat	P/wo
*t*/*P*	-2.73	0.013	-2.42	0.025	-2.25	0.036	-2.39	0.027	-2.39	0.027
Cohen’s *d*/WO	-1.252	180	-1.110	420	-1.032	440	-1.097	440	-1.097	400

WO is the time window. The time window was set to 20 ms.

#### 3.2.2 Spatiotemporal pattern of ERP of SPM (*t*)

**FIGURE 7:**
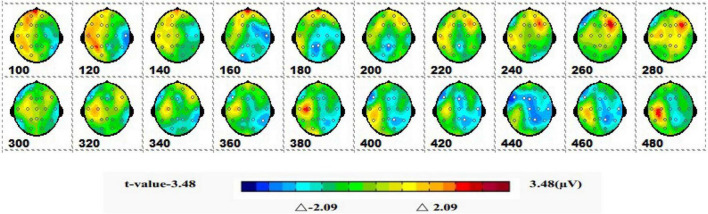
The ERPs of 1-back non-spatial working memory in upright position vs. 1-back non-spatial working memory in seated position were tested by two-tailed paired *T*-test, and the spatio-temporal pattern of SPM (*t*) (0–600 ms) was obtained by means of interpolation in average *T*-value. The time window is set at 20 ms. The colors beyond the 0.05 significant threshold (19) = 2.09 at the two ends of the color scale represent significant regions. The white dots represent the electrode sites with significant differences.

Figure 7 is a topographic map of the SPM (*t*) (0-600 ms) two-tailed paired *T*-test results. The white bright blue locations were divided into thresholds corresponding to *P* = 0.05: *t*(1,19) = ±2.09. The white dots on the topographic map represent electrode sites with significant differences. The 1-back spatial working memory in the upright position and 1-back non-spatial working memory in the seated position were initially shown in the parieto-occipital lobe at 160-200 ms, with a higher mean amplitude in the upright position than in the seated position. After that, significant differences again appeared in the central parietal region between 400 and 460 ms, and the mean amplitude was higher in the sitting position than in the standing position.

### 3.3 Spatiotemporal patterns of average waveform and SPM (*t*) of sitting 1-back spatial working memory vs. sitting 2-back spatial working memory

#### 3.3.1 Average waveform and component analysis

**FIGURE 8:**
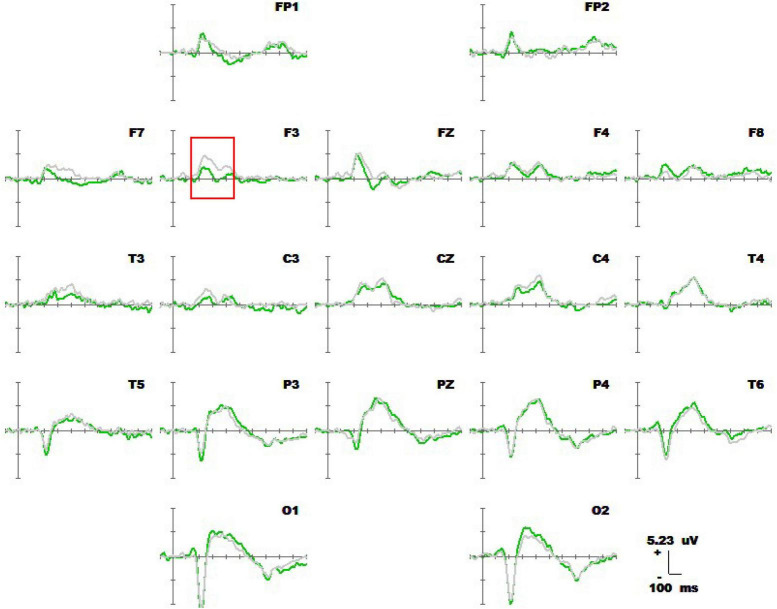
Average waveforms of the ERPs at 19 electrodes (–100 to 600 ms) for 20 subjects performing 1-back spatial working memory (green waveform) while seated vs. 2-back spatial working memory (gray waveform) while seated at 19 electrodes (–100 to 600 ms). The baseline of the ERPs was the average amplitude of the waveforms within 100 ms before the stimulus was presented.

Figure 8 shows the 1-back spatial working memory in the seated position (green waveform) and 2-back spatial working memory in the seated position (gray waveform). There were differences between the two groups in the waveform at the forehead (220-380 ms). At this stage, the average P3a amplitude of the 1-back working memory at electrode F3 was lower than that of the 2-back working memory. The specific statistical data for amplitude differences between the two ERPs at typical electrodes are shown in Table 7. The maximum expression value of the P300 effect was 3.45 μV at 260 ms at electrode F3, which corresponded to an amplitude of 1.90 μV in the 1-back space working memory and 4.35 μV in the 2-back space working memory.

**TABLE 7:** Significant waveform differences in ERP between sitting 1-back space working memory and sitting 2-back space working memory (*n* = 20).

Effect	P3a (F3)		P3a (F3)		P3a (F3)		P3a (F3)		P3a (F3)	
	Stat	P/wo	Stat	P/wo	Stat	P/wo	Stat	P/wo	Stat	P/wo
*t*/*P*	-2.42	0.025	-2.31	0.032	-2.57	0.018	-2.97	0.008	-3.21	0.004
Cohen’s *d*/WO	-1.110	220	-1.060	240	-1.179	260	-0.091	280	-1.473	300

WO is the time window. The time window was set to 20 ms.

#### 3.3.2 Spatiotemporal pattern of ERP of SPM (*t*)

**FIGURE 9:**
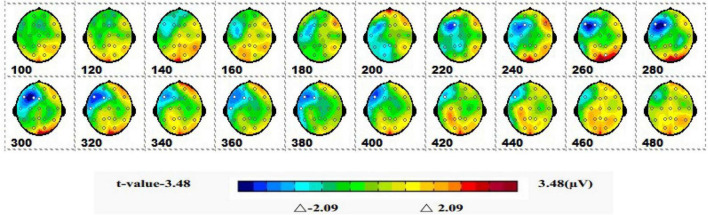
The ERPs of 1-back space working memory in sitting position vs. 2-back space working memory in sitting position were tested by two-tail paired *T*-test, and the spatio-temporal pattern of SPM (*t*) (0–600 ms) was obtained by interpolation in average *T*-value. The time window is set at 20 ms. The colors beyond the 0.05 significant threshold (19) = 2.09 at the two ends of the color scale represent significant regions. The white dots represent the electrode sites with significant differences.

Figure 9 is a topographic map of the SPM (*t*) (0-600 ms) two-tailed paired *T*-test results. The white and bright blue locations were divided into thresholds corresponding to *P* = 0.05: *t*(1,19) = ±2.09. The white dots on the topographic map represent electrode sites with significant differences. The 1-back spatial working memory in the seated position and 2-back spatial working memory in the seated position showed that the average P3a amplitude of the 1-back spatial working memory was lower than that of the 2-back spatial working memory in the prefrontal lobe at 220-380 ms.

### 3.4 Spatiotemporal patterns of average waveform and SPM (*t*) of sitting 1-back spatial working memory vs. sitting 1-back non-spatial working memory

#### 3.4.1 Average waveform and component

**FIGURE 10:**
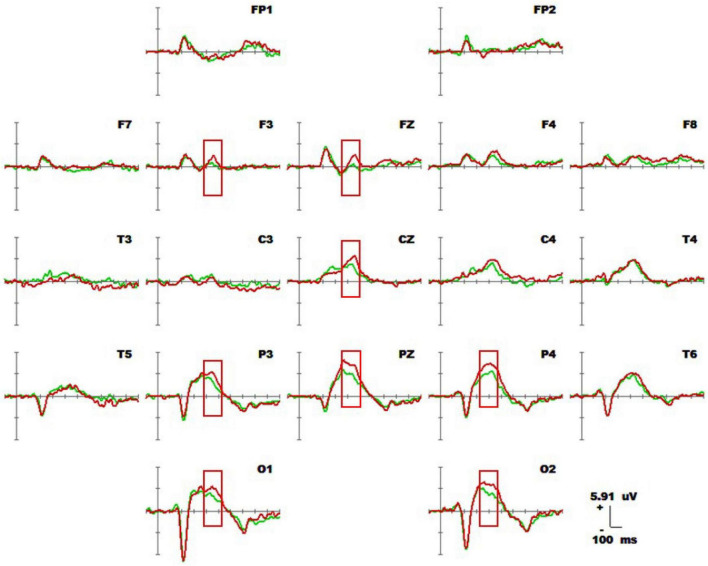
Average waveforms of 1-back spatial working memory (green waveform) vs. 1-back non-spatial working memory (red waveform) for 20 subjects sitting down at 19 electrodes (–100 to 600 ms). The baseline of the ERPs was the average amplitude of the waveforms within 100 ms prior to stimulus presentation.

Figure 10 shows the 1-back spatial working memory (green waveform) and 1-back non-spatial working memory (red waveform) in the seated position. The readings for the occipital lobe, parietal lobe, forehead, and central region (360-460 ms) are represented in the waveforms of the two groups. At this stage, the average amplitudes of the P300 1-back spatial working memory at electrodes Fz, F3, P4, P3, Pz, Cz, O1, and O2 were lower than those of P300 in the 1-back non-spatial working memory. The specific statistical data for the amplitude differences between the two ERPs at typical electrodes are shown in Table 8. The maximum expression value of the P300 effect was 3.38 μV at 460 ms at the P4 electrode, and the corresponding amplitude of the 1-back spatial working memory was 3.15 μV, while that of the 1-back non-spatial working memory was 6.53 μV.

**TABLE 8:** Differences in ERP between 1-back visual spatial working memory and 1-back visual non-spatial working memory in sitting position showing significant waveform effect (*n* = 20).

Effect	P300 (P4)		P300 (Pz)		P300 (Pz)		P300 (P3)		P300 (P4)	
	**Stat**	**P/wo**	**Stat**	**P/wo**	**Stat**	**P/wo**	**Stat**	**P/wo**	**Stat**	**P/wo**
*t*/*P*	-2.22	0.038	-2.44	0.024	-2.77	0.012	-2.62	0.016	-2.68	0.014
Cohen’s *d*/WO	-1.019	360	-1.120	380	-1.271	440	-1.202	440	-1.230	460
**Effect**	**P300 (O1)**		**P300 (O2)**		**P300 (Fz)**		**P300 (Cz)**		**P300 (F3)**	
	**Stat**	**P/wo**	**Stat**	**P/wo**	**Stat**	**P/wo**	**Stat**	**P/wo**	**Stat**	**P/wo**
*t*/*P*	-2.36	0.029	-2.14	0.045	-2.15	0.044	-2.51	0.020	-2.42	0.025
Cohen’s *d*/WO	-1.083	460	-0.981	460	-0.982	440	-1.152	460	-1.110	440

WO is the time window. The time window was set to 20 ms.

#### 3.4.2 Spatiotemporal patterns of SPM (*t*)

**FIGURE 11:**
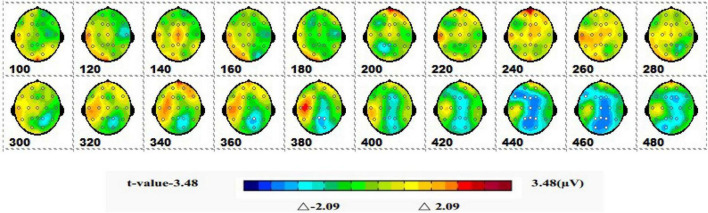
The ERPs of 1-back spatial working memory in seated vs. 1-back non-spatial working memory in seated were tested by the two-tailed paired *T*-test, and the spatio-temporal pattern of SPM (*t*) (0–600 ms) was obtained by interpolation within the mean *T*-value. The time window is set at 20 ms. The colors beyond the 0.05 significant threshold (19) = 2.09 at the two ends of the color scale represent significant regions. The white dots represent the electrode sites with significant differences.

Figure 11 shows a topographic map of the SPM (*t*) (0-600 ms) two-tailed paired *T*-test results. The white and bright blue locations are divided into *t* thresholds corresponding to *P* = 0.05: *t*(1,19) = ±2.09. The white dots on the topographic map represent electrode sites with significant differences. The 1-back spatial working memory in sitting position and 1-back non-spatial working memory in sitting position were displayed in the occipital lobe, parietal lobe, and central region (360-460 ms), and the average P3b amplitude of 1-back spatial working memory was lower than that of 1-back non-spatial working memory.

## 4 Discussion

N is a key variable of the n-back paradigm. Many studies have investigated the impact of cognitive load on working memory updates by changing the parameter N. When performing visuospatial and non-spatial working memory in the upright position, the response time was extended, and the accuracy decreased with an increase in task difficulty. These research results are consistent with those of Luo et al. (2015) and Reed et al. (2017). In visuospatial working memory, there was no significant difference in response time or accuracy with an increase in task difficulty. In the case of visual non-spatial working memory, the response time increased with the difficulty of the task. There may be sufficient cognitive resources in the sitting position but limited cognitive resources in the upright position, resulting in increased cognitive difficulty and, thus, a significant difference in the behavioral data of reaction time and accuracy. Brain imaging studies have shown that the degree of prefrontal cortex activation increases with an increase in working memory load (Herff et al., 2013). As the difficulty of the tasks increased in this study, as shown in Figure 9, the P3a of visuospatial working memory at F3 on the forehead increased. With an increase in cognitive difficulty, increasingly, more attention was required to complete tasks of low cognitive difficulty, which is consistent with the research results of Herff et al. (2013). Mccarthy and Donchin (1981) formed similar research conclusions. When studying the ERP of typical working memory, he found that the P300 amplitude increased with an increase in task load (Mccarthy and Donchin, 1981; Hagen et al., 2006). P300 volatility is an index that reflects the resources allocated to different tasks. The more resources are allocated, the greater the volatility (Kok, 2010).

In the upright position, the reaction time of 1-back visuospatial working memory was longer than that of visual non-spatial working memory, but the difference in accuracy was not statistically significant. The reaction time of 2-back visuospatial working memory was longer than that of visual non-spatial working memory, while accuracy was greater for visual non-spatial working memory, with both showing statistical significance. In the seated position, the reaction time of 1-back spatial visual working memory was longer than that of non-spatial visual working memory, but the accuracies were comparable. There were no significant differences in response time or accuracy between 2-back visual empty working memory and visual non-spatial working memory. In Figure 11, brain imaging studies have shown that both 1-back spatial working memory and 1-back non-spatial working memory in the seated position displayed activity in the occipital lobe, parietal lobe, and central region (360-460 ms), and the average P3b amplitude of 1-back spatial working memory was lower than that of 1-back non-spatial working memory. P300 volatility is an index that reflects the resources allocated to different tasks. The more resources are allocated, the greater the volatility (Kok, 2010). This indicates that non-spatial visual memory consumes more cognitive resources than spatial memory, and the difference in the coding stage between the two is mainly in the parieto-occipital lobe.

Compared with in the seated position, participants in the upright position had statistically different reaction times and accuracies in the 1-back visuospatial task, while reaction time in the 1-back visuospatial task in the upright position was faster and more accurate than that in the seated position. In the 1-back visual non-spatial task, those in the upright position had faster reaction speed, and the difference was statistically significant, but there was no difference in accuracy. For the 2-back visuospatial and 2-back visuo non-spatial tasks, there were no significant differences in accuracy or reaction time between those in the sitting position and upright position. The upright posture promoted better responses in the simple visuospatial and non-spatial tasks. As the difficulty increased, posture had no significant effect on the participants ability to perform the two tasks. Because of the time limits of this study, only 1-back and 2-back tests were conducted; however, further 3-back tests can be carried out for comparison. Lacour et al. (2009) proposed that there is an “inverted U” relationship between arousal level and behavioral control.

1-Back spatial working memory was performed in the seated position, there were two stages of differences in waveforms between the two groups. The first stage was N1 (160-180 ms) in parieto-occipital lobe. During this time, the amplitude of N1 at electrodes O1 and P3 in those in the standing position was higher than that in the lower position. The second stage was in the occipital lobe (280-360 ms). In this stage, the amplitude of P300 at the O1 and O2 electrodes in the upright position was lower than that in the seated position. Similar to the results of previous studies by Quant et al. (2004) and Little and Woollacott (2015), the N1 effect in the frontal, central, and parietal lobes was triggered under different body positions, but in this study, the N1 effect was also triggered in the occipital lobe. N1 is considered to be related to selective attention, and with attention decreases, the N1 amplitude decreases with it (Hawkes, 2003). Studies have found that N1 is positively correlated with attention, when attention decreases, the amplitude of N1 also decreases (Peters et al., 2003; Herff et al., 2013). Finnigan observed that attention decreased and N1 amplitude decreased with perceptual stimulation, distraction, and inattention (Kutas and Hillyard, 1980). As shown in Figure 7, visuospatial working memory performed in the upright position had a higher N1 amplitude than that performed in the seated position. This is consistent with the findings of other behavioral studies, and behavioral data analysis shows that the reaction speed is improved for subjects in the upright position. The results of behavioral and EEG studies indicate this effect is related to the enhancement of attention in the upright position. When performing 1-back visual non-spatial tasks, the N1 effect was also induced in the upright and sitting positions, and the N1 amplitude in the visual non-spatial tasks while upright was larger than that in the seated position, which is roughly consistent with the behavioral index, i.e., the response speed was faster in the upright position. There was no difference in N1 amplitude between spatial working memory and non-spatial working memory.

The amplitude of P3b in the occipital lobe was lower in the standing position than the seated position, which may have been because controlling an upright posture consumes more cognitive resources, leading to a decline in the waveform related to P3b information-encoding in the occipital lobe. Although postural control promoted subjects’ attention, it still competed with visuospatial cognitive tasks for central information processing resources. When 1-back non-spatial working memory was performed in the upright position and 1-back non-spatial working memory was performed in the seated position, there were two stages of differences in waveform between the two groups. The first stage was N1 (160-180 ms) in the parieto-occipital lobe. During this time, the amplitudes of N1 of those in the standing position at electrodes O1, P3, and Pz were higher than those in sitting position. The second stage was in the occipital lobe (400-460 ms). In this stage, the P300 amplitudes at the Fz, F3, P4, Pz, and Cz electrodes of those in the upright position were lower than those in the seated position. The results show that maintaining an upright posture also competes with visual non-spatial cognitive tasks for central information-processing resources. We demonstrated that upright postural control enhances early selective attention but interferes with central memory encoding, thus confirming that postural control and visuospatial and non-spatial working memory share brain regions and compete with each other.

In this study, ERP demonstrated that upright posture control competed for resources with cognitive tasks. The amplitude of both visuospatial and non-spatial working memory P300 increased with an increase in working memory load. The amplitude of P300 in the visual spatial working memory task was lower than that in the visual non-spatial working memory task. Due to the time limitations of this study, only 1-back and 2-back tests were conducted, but 3-back tests can be further carried out for comparison in the future.

## Data availability statement

The datasets presented in this study can be found in online repositories. The names of the repository/repositories and accession number(s) can be found in the article/supplementary material.

## Ethics statement

The studies involving humans were approved by the Medical Ethics Committee of The First Affiliated Hospital of Guangdong Pharmaceutical University. The studies were conducted in accordance with the local legislation and institutional requirements. The participants provided their written informed consent to participate in this study. Written informed consent was obtained from the individual(s) for the publication of any potentially identifiable images or data included in this article.

## Author contributions

SS: Formal analysis, Writing – original draft, Writing – review & editing. FH: Supervision, Writing – original draft. LC: Methodology, Writing – original draft. CS: Project administration, Software, Writing – original draft. JL: Methodology, Writing – original draft. ZC: Methodology, Resources, Writing – original draft, Writing – review & editing. WC: Data curation, Formal analysis, Project administration, Software, Writing – original draft, Writing – review & editing.
